# Supplementary material for: Four New Unusual Pentacyclic Triterpenoids from the Roots of Jasminum sambac (L.) Ait
Source: Molecules. 2023 Jun 29;28(13):5097. doi: 10.3390/molecules28135097 (PMC10343477; doi:10.3390/molecules28135097)
Supplement: Supplementary file 1 [file molecules-28-05097-s001.zip › molecules-2430080-supplementary.pdf]

Supporting Information

Four new unusual pentacyclic triterpenoids from the roots of *Jasminum sambac* (L.) Ait

Olagoke Zacchaeus Olatunde [1,3], Jianping Yong [2],* and Canzhong Lu [1,2,3],*
[1] Fujian Institute of Research on the Structure of Matter, Chinese Academy of Sciences, Fuzhou 350002, China; olatunde@fjirsm.ac.cn
[2] Xiamen Institute of Rare-Earth Materials, Chinese Academy of Sciences, Xiamen 361021, China
[3] University of Chinese Academy of Sciences, Beijing 100049, China
* Correspondence: jpyong@fjirsm.ac.cn (J.Y.); czlu@fjirsm.ac.cn (C.L.);
Tel.: +86-591-63173162 (J.Y.); +86-591-63173355 (C.L.)

Plant material:

The roots of *J. sambac* were collected from Ningde county of Fujian Province in Autumm of 2020.

The detailed Isolation procedures:

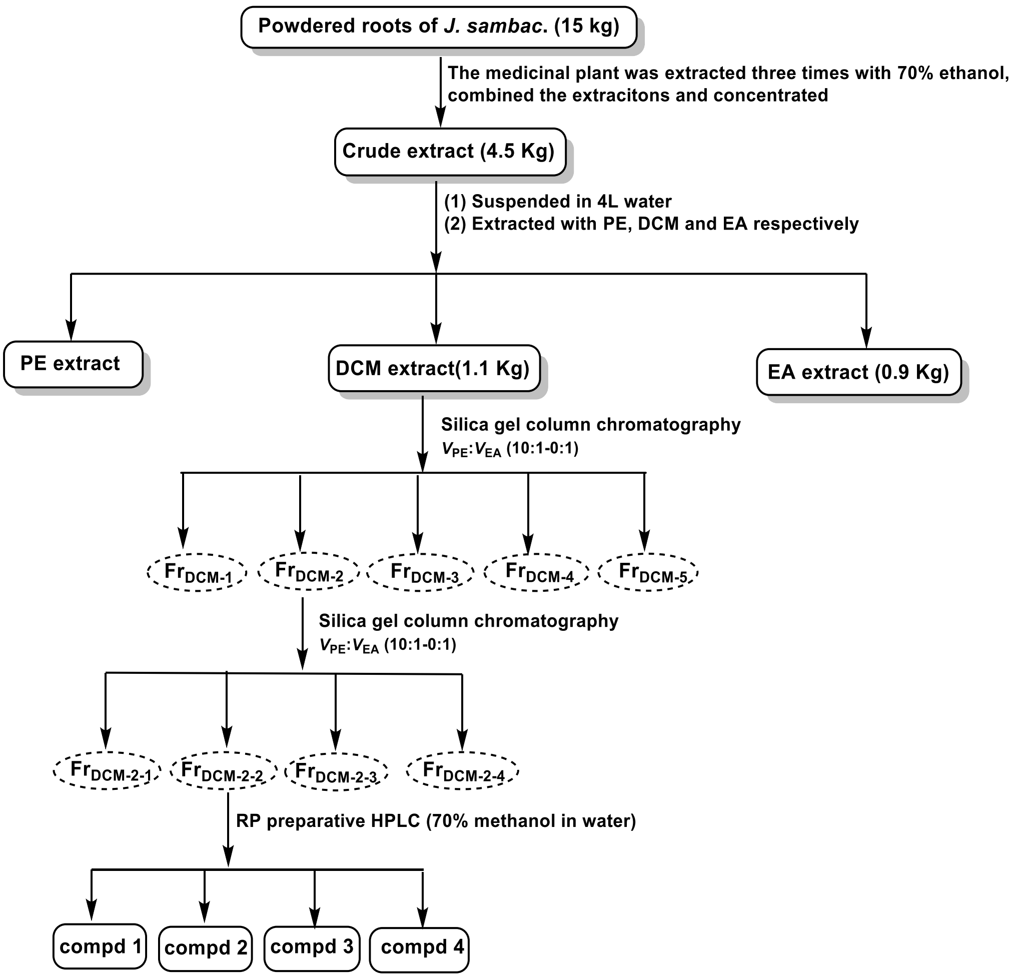

Figure S1. The concise isolation procedures

The air-dried roots of *J. sambac* (15.0 kg) were soaked in 70% $V_{ethanol}/V_{water}$ solution. After a month, the extract was filtered and the filtrate was concentrated under vacuum to obtain a crude extract (4.5 kg).

The crude extract (4.5 kg) was suspended in 4000 mL water, then extracted with

petroleum ether (PE) (3×2L), dichloromethane (DCM) (3×2L), and ethyl acetate (EA) (3×2L) respectively. The DCM and EA layers were concentrated under reduced pressure to obtain 1.1kg and 0.9kg extracts respectively.

The **DCM extract** (1.1 kg) was fractionated to silica gel column chromatography with a step-wise gradient system PE/EA: (V_{PE}:V_{EA}, 10:1 to 0:1) to obtain different fractions: **1 fraction** was obtained from V_{PE}:V_{EA}, 4:1, **4 fractions** from V_{PE}:V_{EA}, 2:1, **5 fractions** from V_{PE}:V_{EA}, 1:1, and **6 fractions** were obtained using EA as eluent. **Five fractions** (**DCM-Fr_1-Fr_5**) were obtained after similar fractions were selected and combined through the simple TLC analysis together with with HPLC. DCM-**Fr_2** **(205g)** was isolated with silica gel column chromatography, eluting with stepwise gradient system of V_{PE}:V_{EA}(10:1 to 0:1) to yield **four subfractions (DCM-**$Fr_{2\text{-}1}$-$Fr_{2\text{-}4}$). DCM-**$Fr_{4\text{-}2}$** was selected to be isolated and purified by preparative RP-HPLC using MeOH/H_2O (70:30: V/V) to obtain compounds **1-4**

Structural information:

Chemical structure, XRD and NMR spectrum of Compound 1

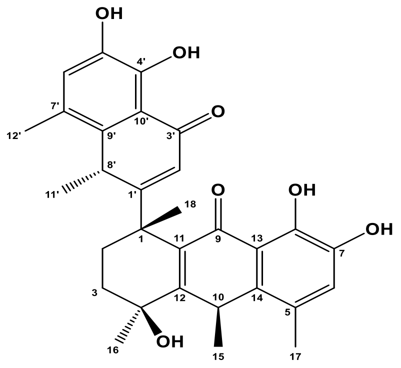

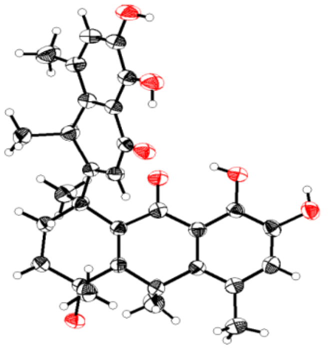

Compound **1:** Golden yellow solid, M.p 61-62 ºC, HPLC purity: 96.596 %, retention time:17.067 min. Crystal Data for compound **1**: orthorhombic, space group $P2_12_12_1$ (no. 19), a = 7.8148(3) Å, b = 13.1614(6) Å, c = 25.3786(11) Å, V = 2610.28(19) Å^3, Z = 4, T = 272.00 K, μ(Mo Kα) = 0.091 mm^{-1}, $Dcalc$ = 1.284 g/cm^3,

22104 reflections measured ($5.454° \leq 2\theta \leq 54.36°$), 5766 unique ($R_{int}$ = 0.0704, R_{sigma} = 0.0650) which were used in all calculations. The final R_1 was 0.0536 (I > 2σ(I)) and wR_2 was 0.1512.

Table S1. Crystal data and structure refinement for compound 1

Identification code	Compound **1**
Empirical formula	$C_{30}H_{32}O_7$
Formula weight	504.55
Temperature/K	272.00
Crystal system	orthorhombic
Space group	$P2_12_12_1$
a/Å	7.8148(3)
b/Å	13.1614(6)
c/Å	25.3786(11)
α/°	90
β/°	90
γ/°	90
Volume/Å^3	2610.28(19)
Z	4
ρ_{calc}g/cm^3	1.284
μ/mm^{-1}	0.091
F(000)	1072.0
Crystal size/mm^3	0.12 × 0.08 × 0.03

Radiation	MoKα (λ = 0.71073)
2Θ range for data collection/°	5.454 to 54.36
Index ranges	$-10 \le h \le 10$, $-14 \le k \le 16$, $-32 \le l \le 32$
Reflections collected	22104
Independent reflections	5766 [R_{int} = 0.0704, R_{sigma} = 0.0650]
Data/restraints/parameters	5766/0/345
Goodness-of-fit on F^2	1.072
Final R indexes [I>=2σ (I)]	R_1 = 0.0536, wR_2 = 0.1258
Final R indexes [all data]	R_1 = 0.0950, wR_2 = 0.1512
Largest diff. peak/hole / e $Å^{-3}$	0.38/-0.29
Flack parameter	-0.6(7)

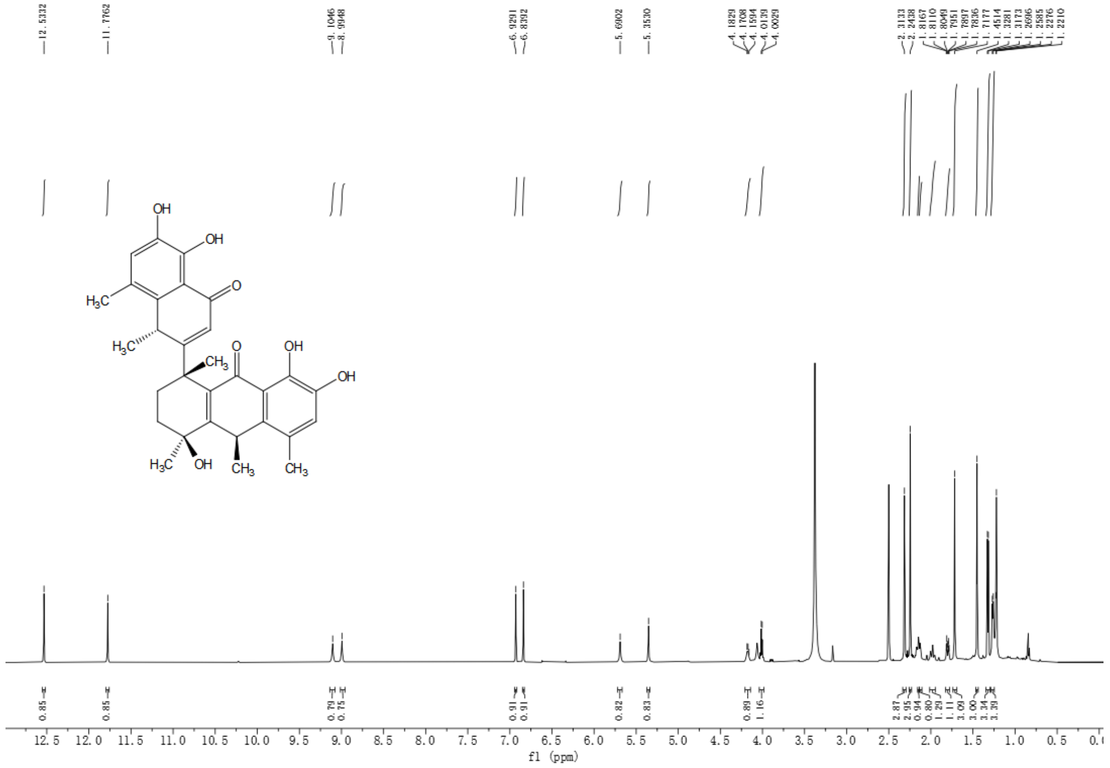

Figure S2: ^{1}H NMR spectrum of compound **1** in DMSO-d_6

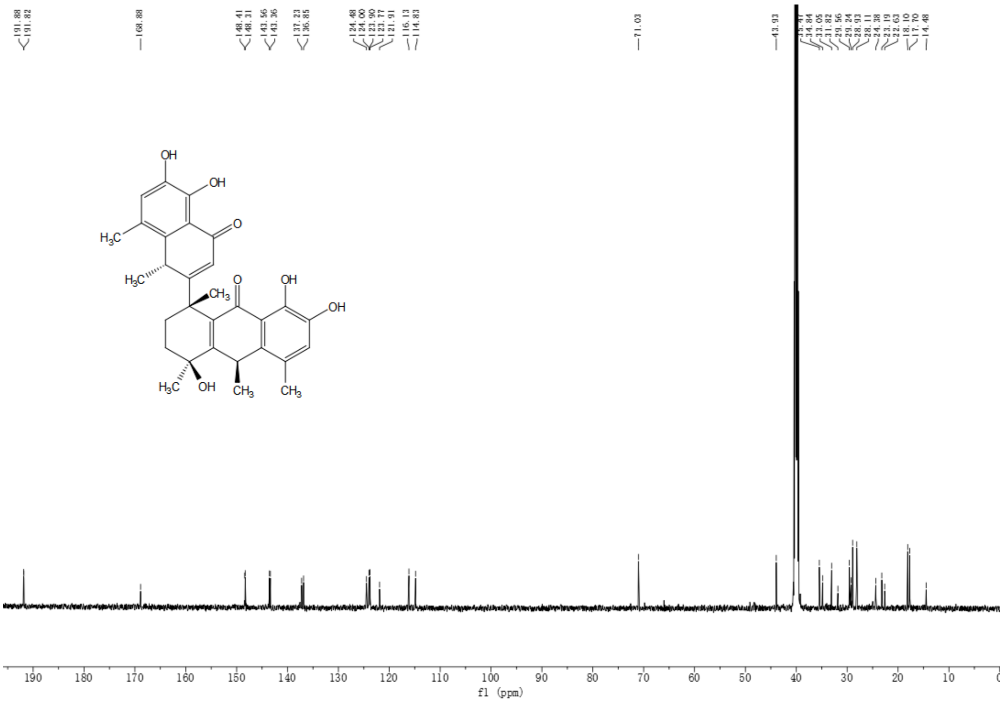

Figure S3: ^{13}C NMR spectrum of compound **1** in DMSO-d_6

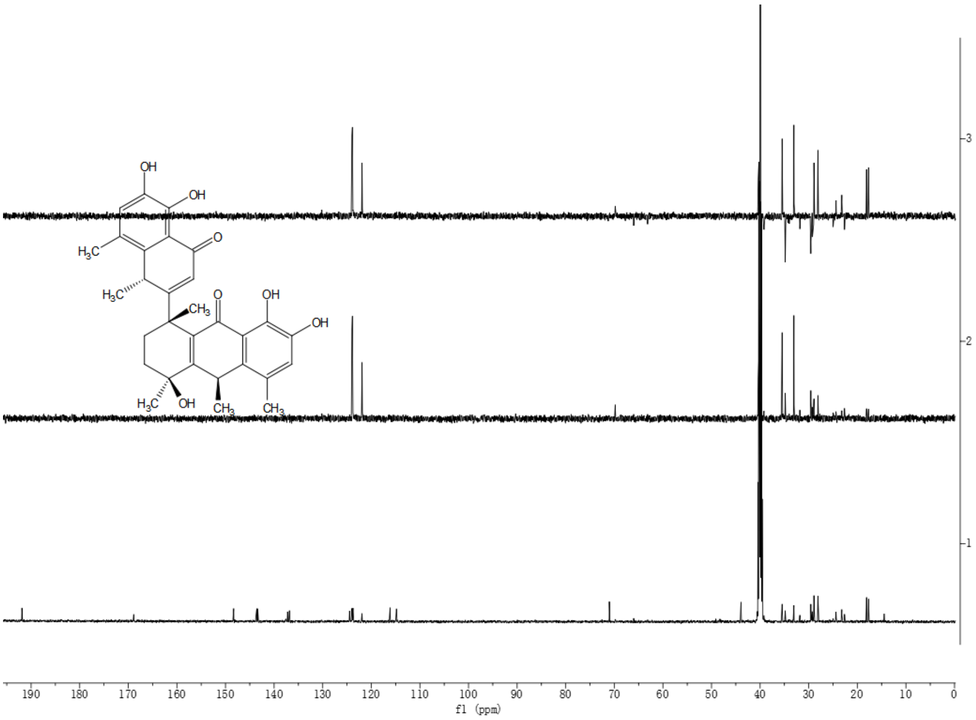

Figure S4: DEPT spectra of compound **1** in DMSO-d_6

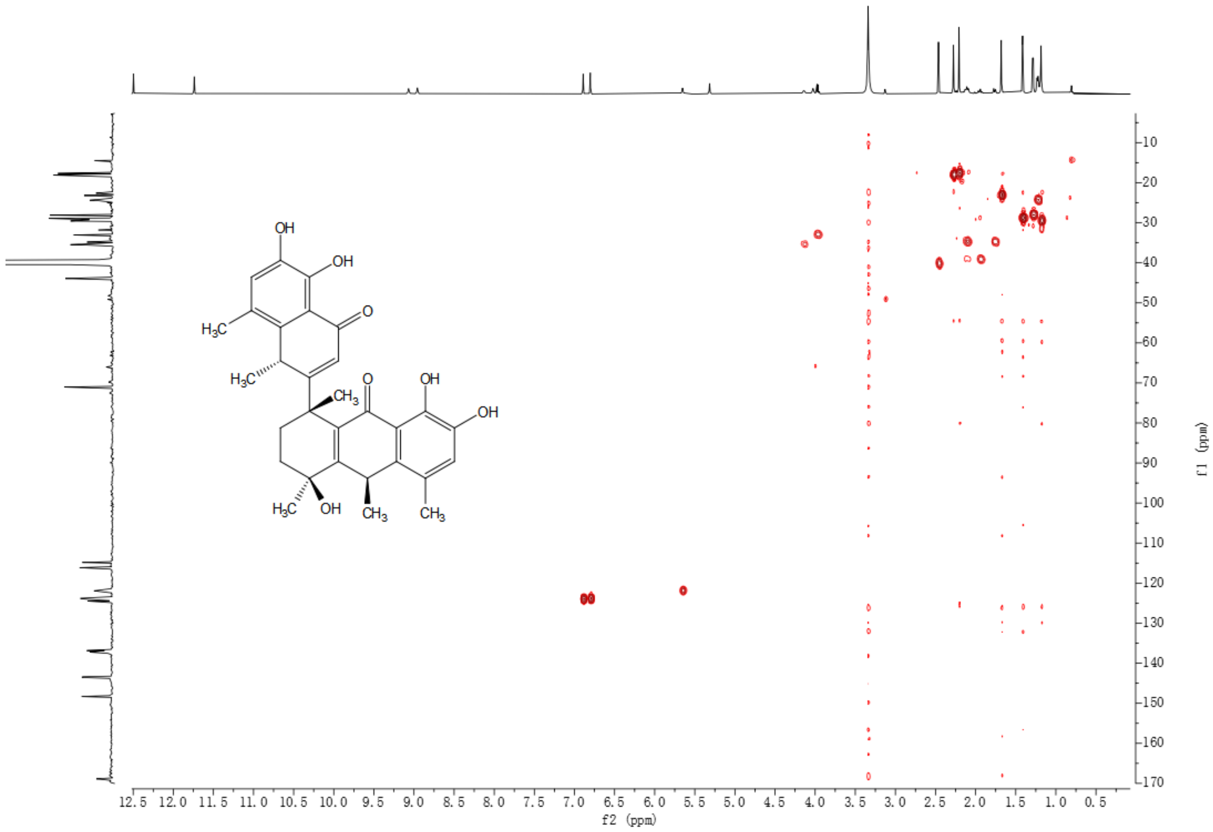

Figure S5: HMQC spectrum of compound **1** in DMSO-d_6

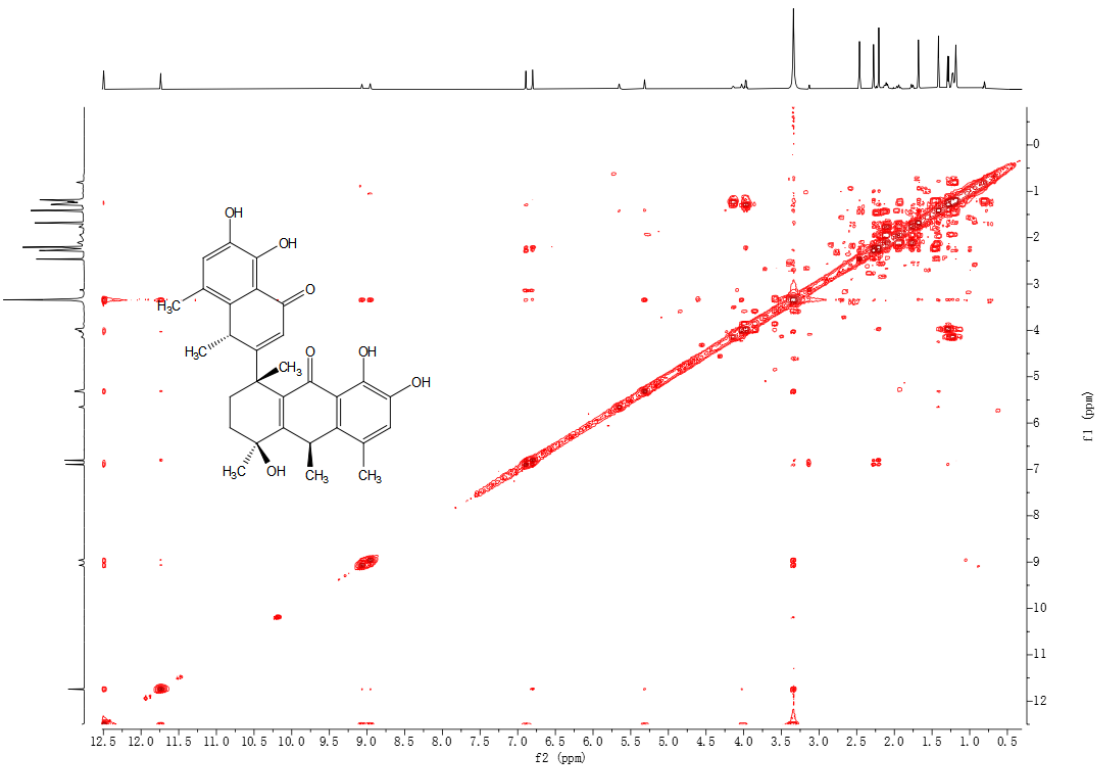

Figure S6: ^1H-^1H COSY spectrum of compound **1** in DMSO-d_6

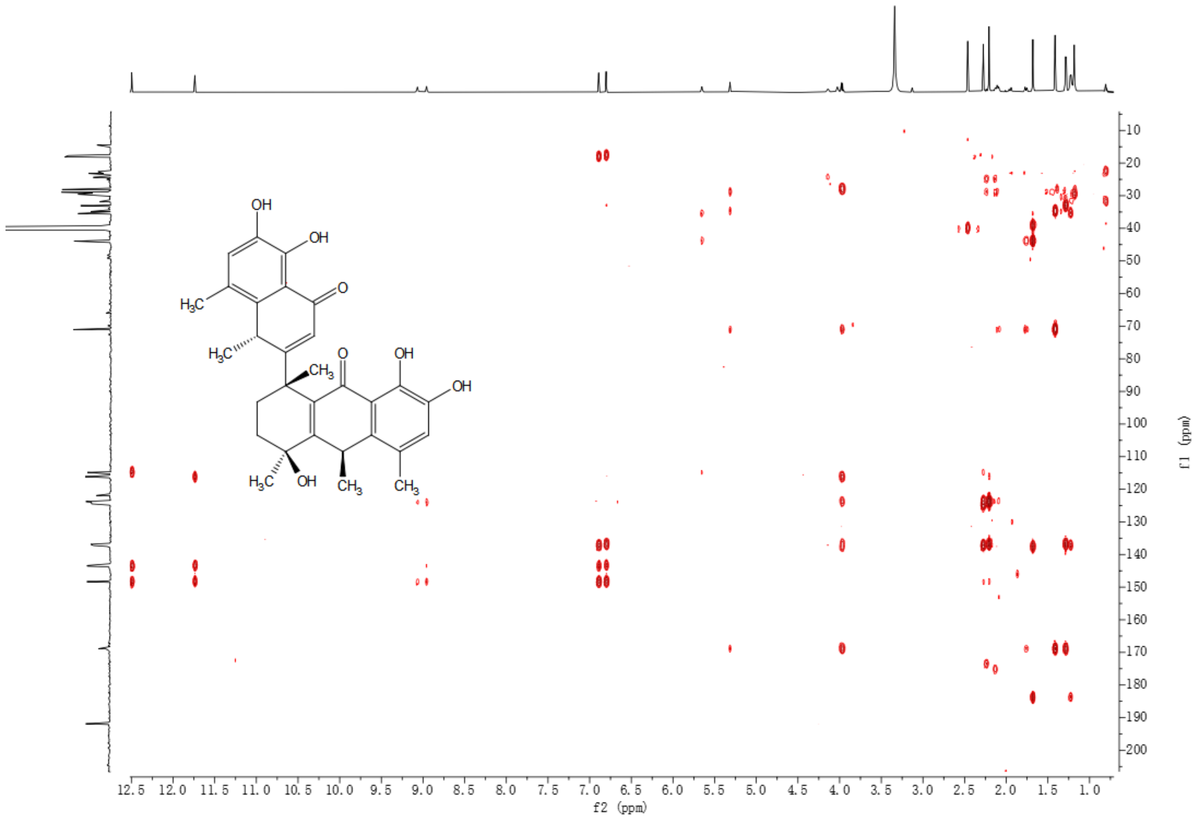

Figure S7: HMBC spectrum of compound **1** in DMSO-d_6

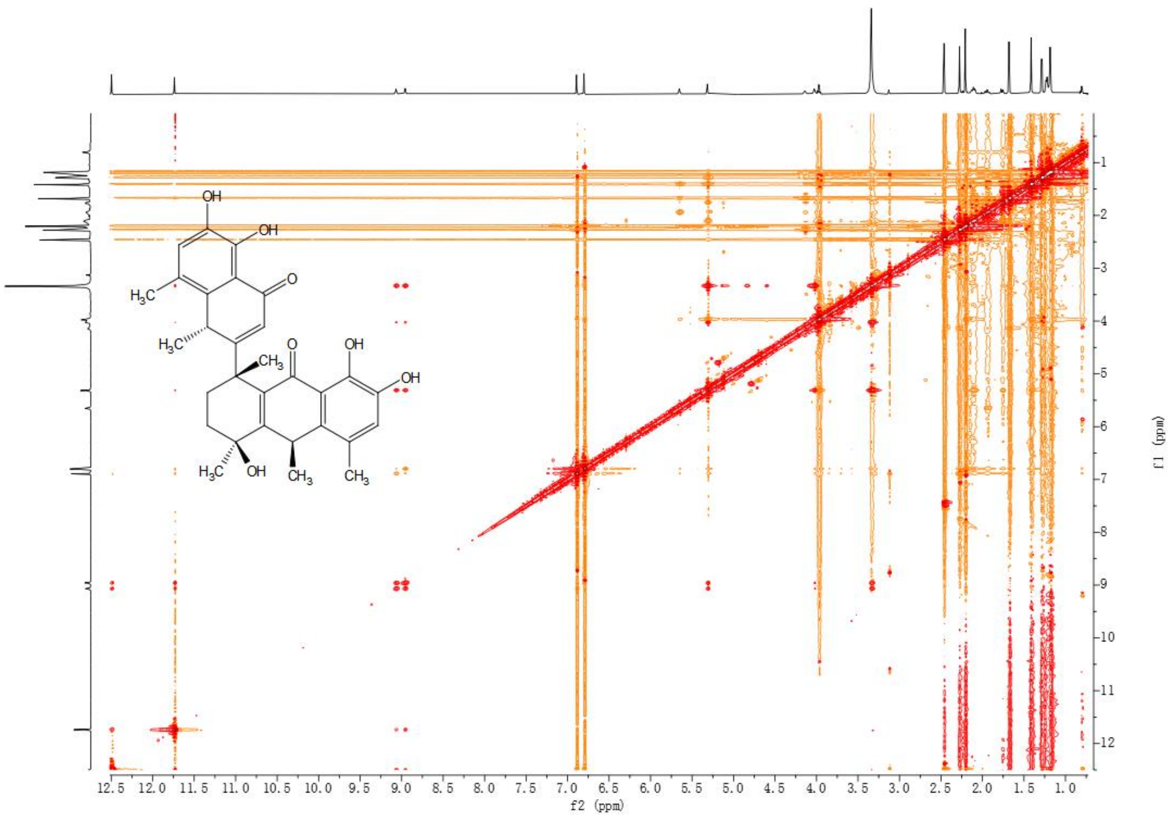

Figure S8: ROESY spectrum of compound **1** in DMSO-d_6

Chemical structure, and NMR date of Compound 2

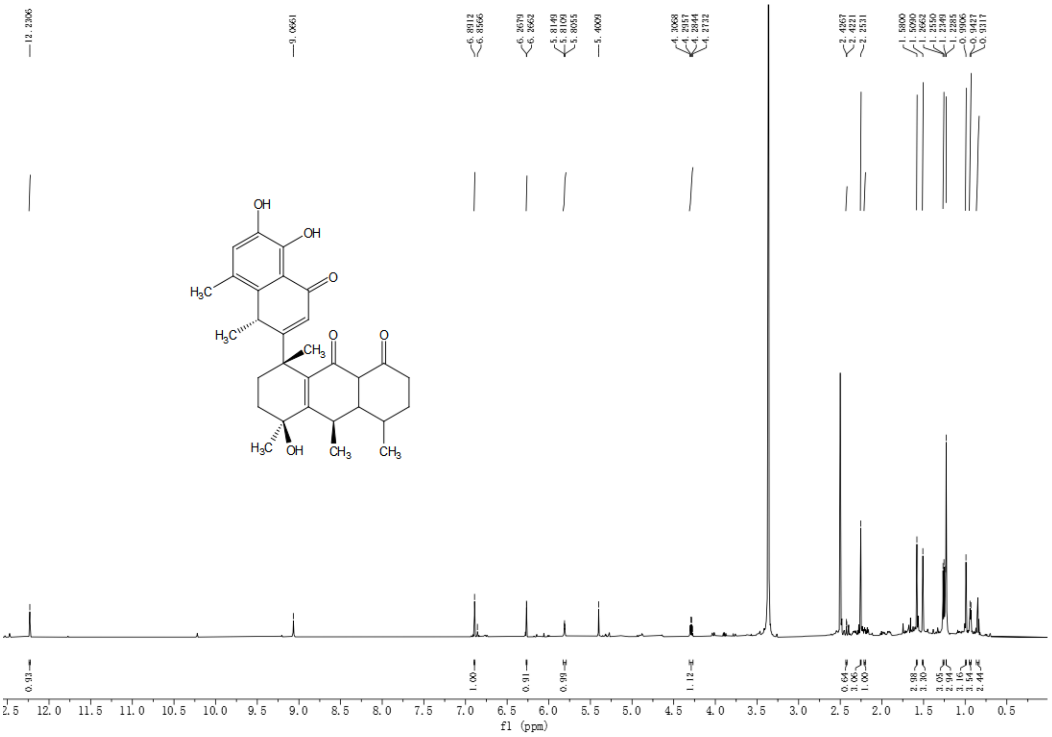

Figure S9: ^{1}H NMR spectrum of compound **2** in DMSO-d_6

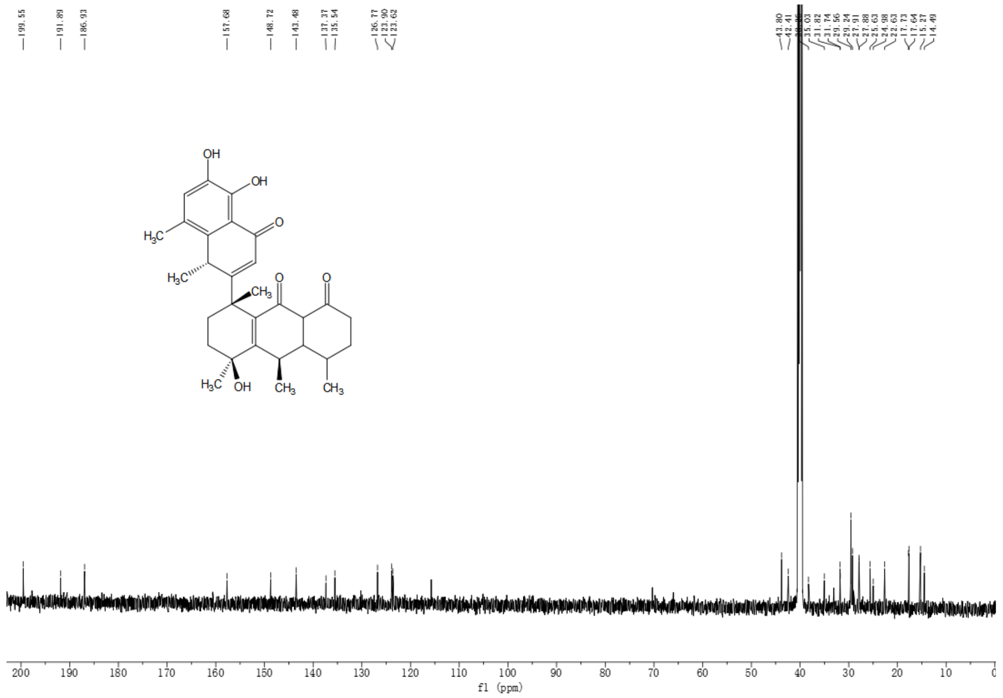

Figure S10: ^{13}C NMR spectrum of compound **2** in DMSO-d_6

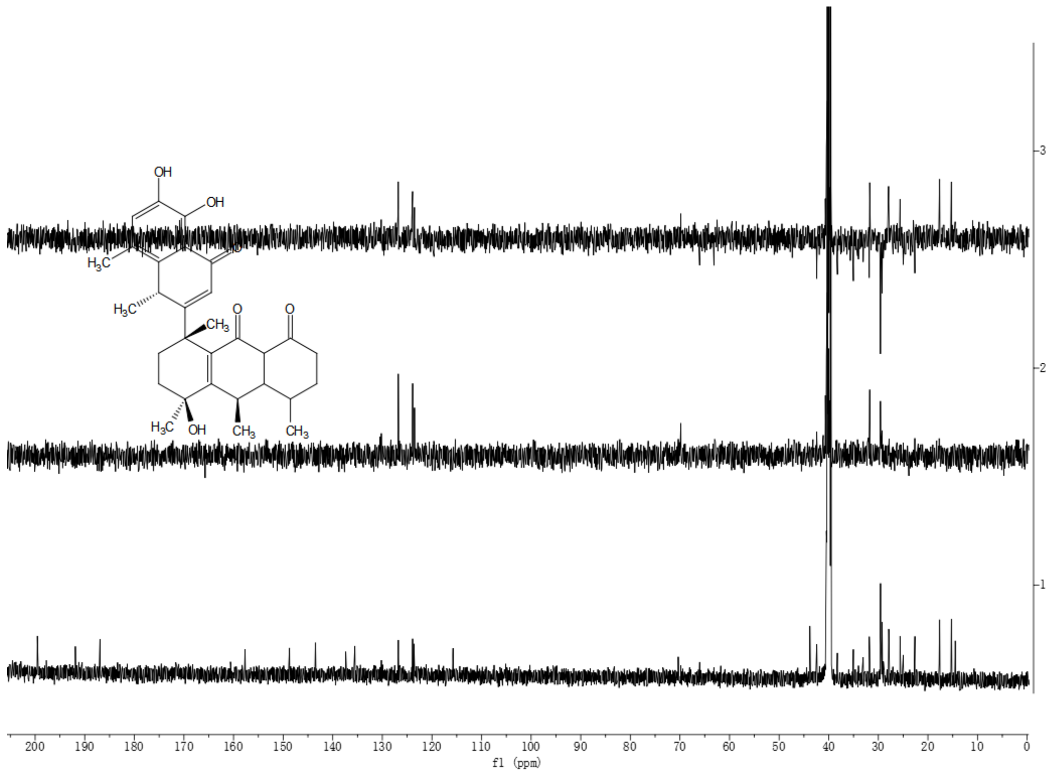

Figure S11: DEPT spectra of compound **2** in DMSO-d_6

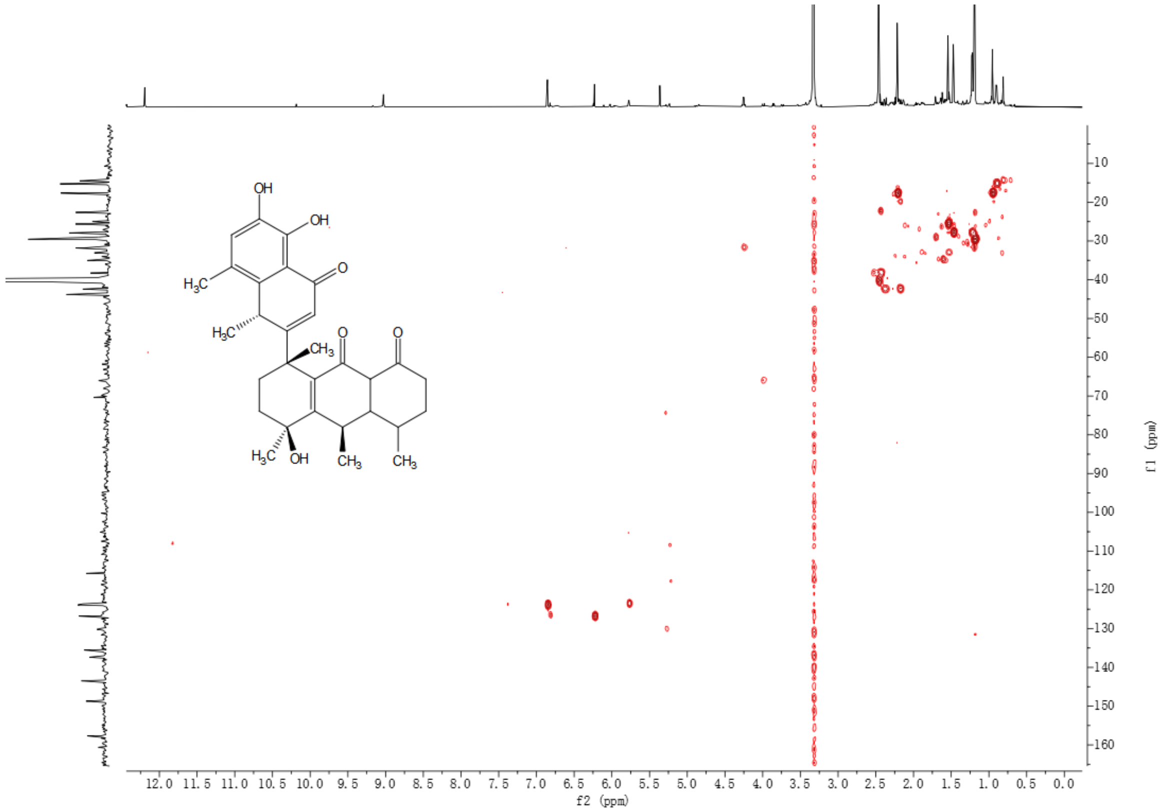

Figure S12: HMQC spectrum of compound **2** in DMSO-d_6

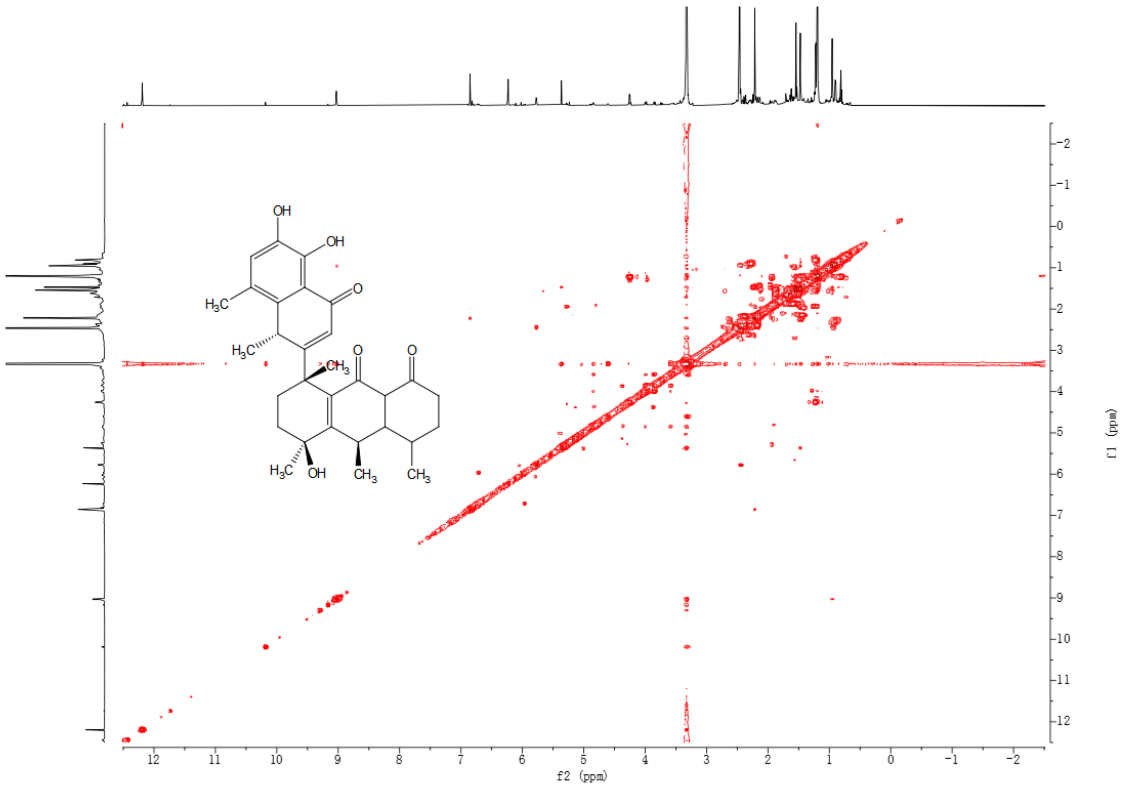

Figure 13: ^{1}H-^{1}H COSY spectrum of compound **2** in DMSO-d_6

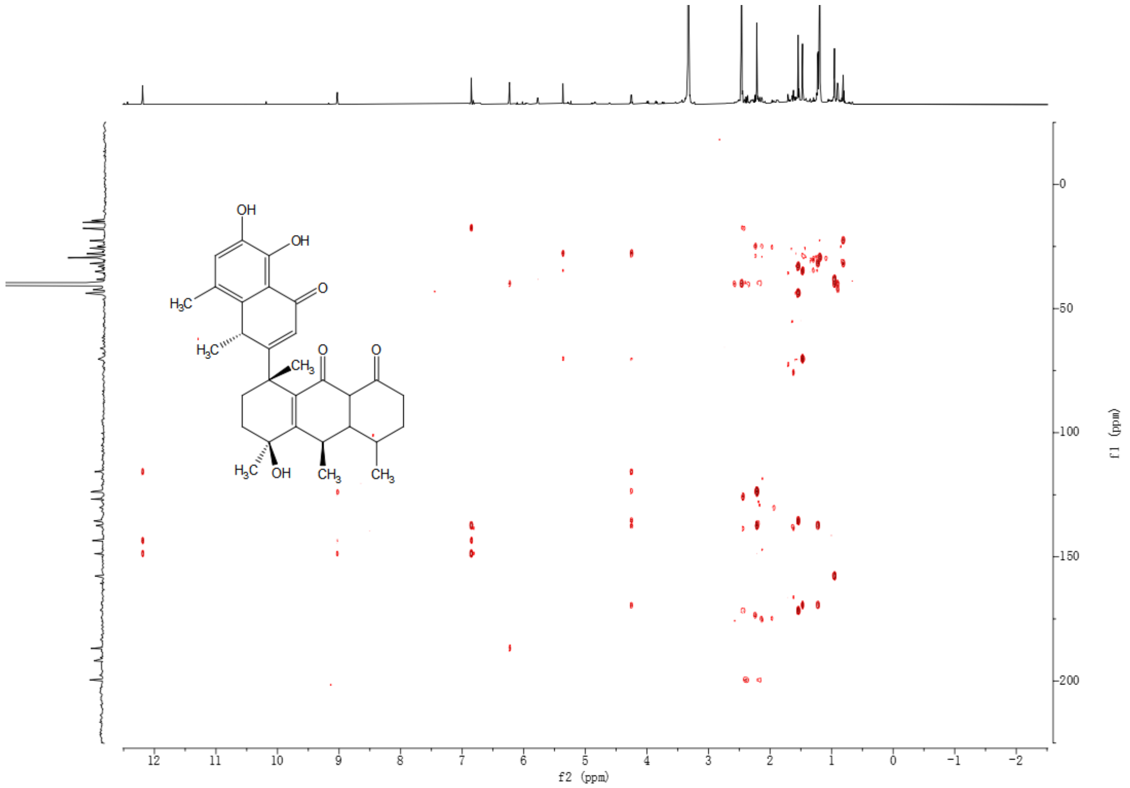

Figure S14: HMBC spectrum of compound **2** in DMSO-d_6

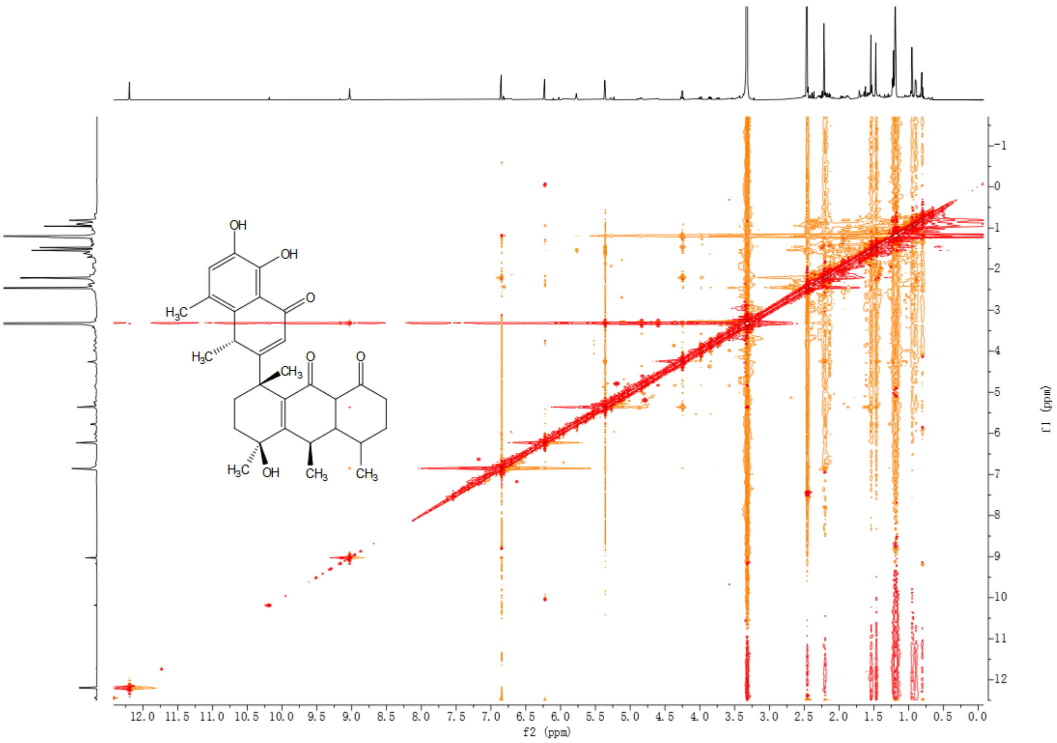

Figure S15: ROESY spectrum of compound **2** in DMSO-d$_6$

Chemical structure, and NMR spectrum of Compound 3

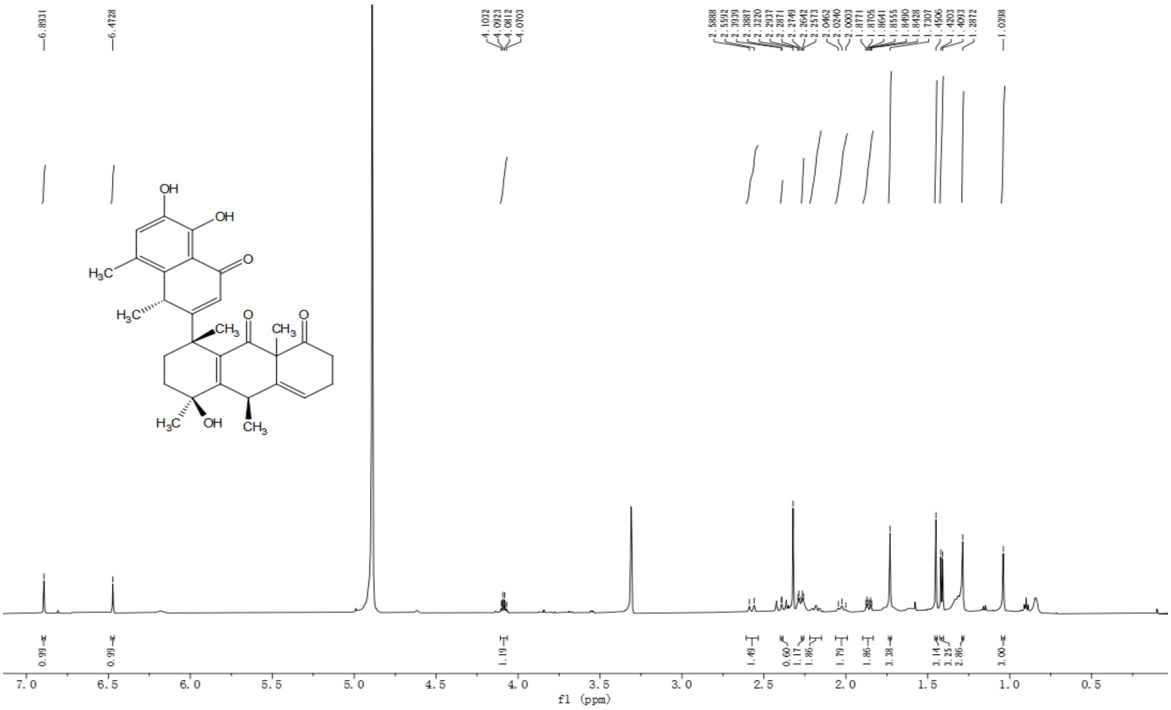

Figure S16: ^{1}H NMR spectrum of compound **3** in CD_3OD

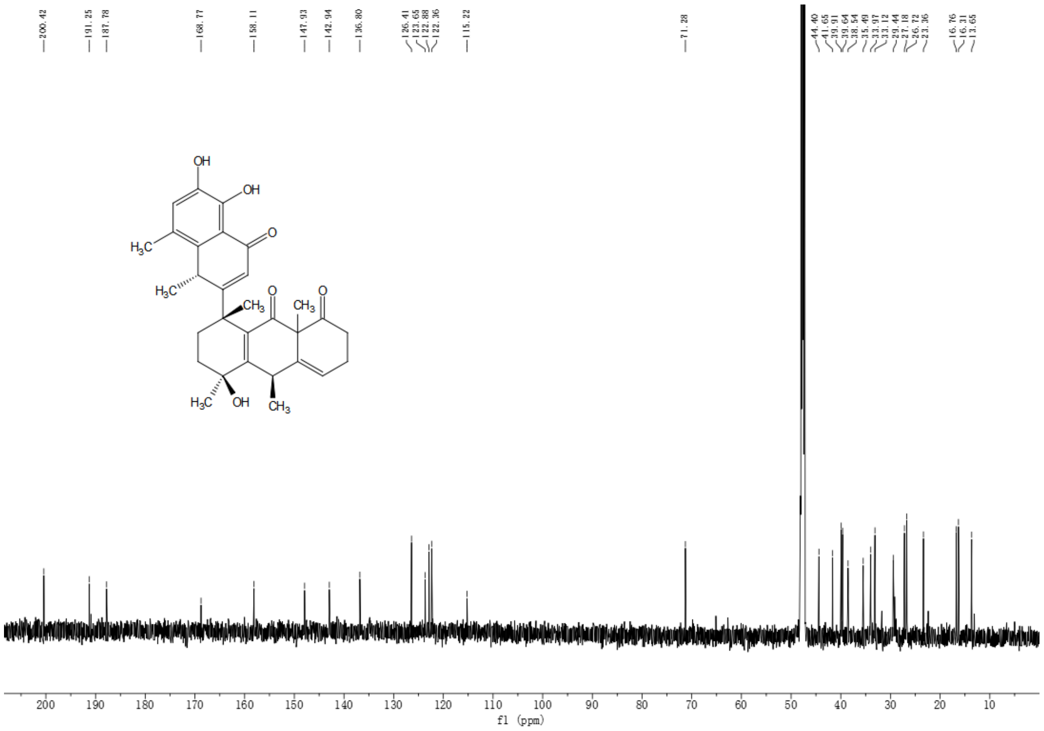

Figure S17: ^{13}C NMR spectrum of compound **3** in CD_3OD

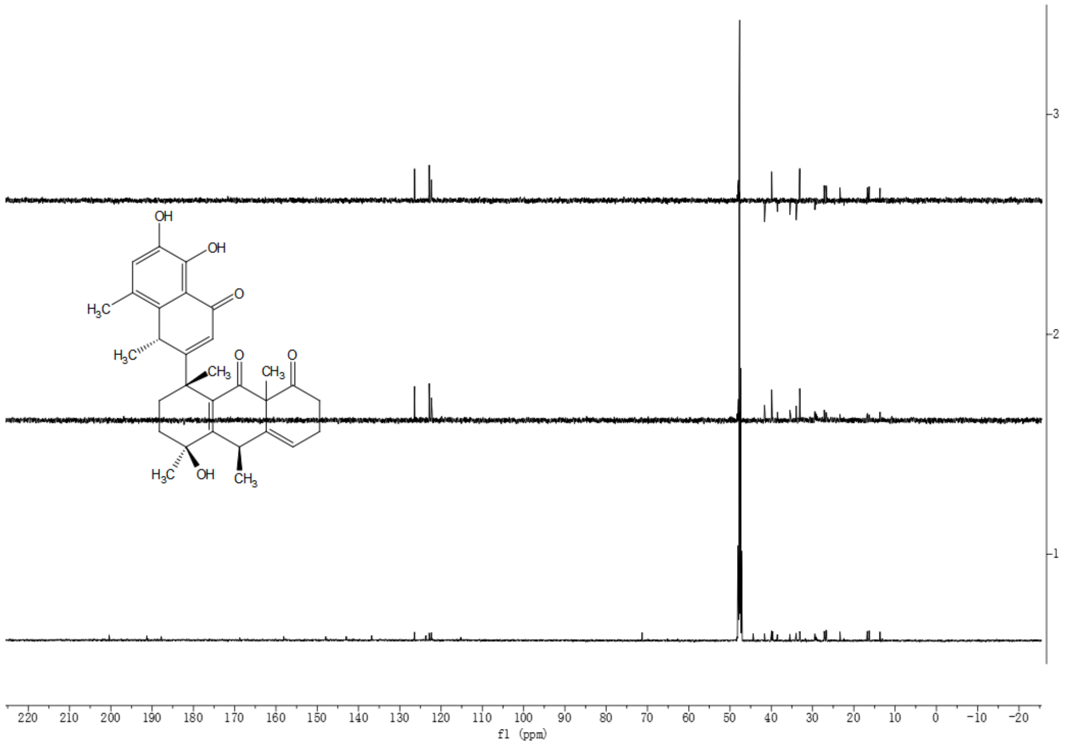

Figure S18: DEPT spectra of compound **3** in CD_3OD

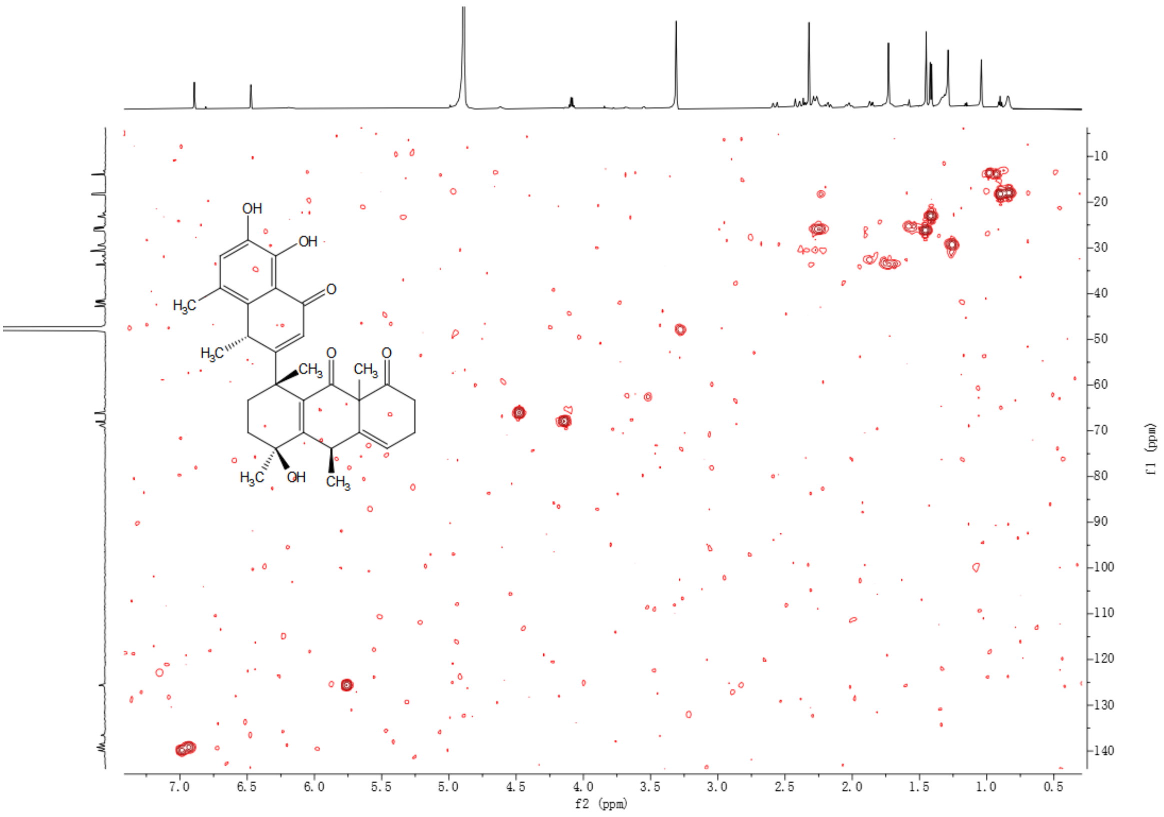

Figure S19: HMQC spectrum of compound **3** in CD_3OD

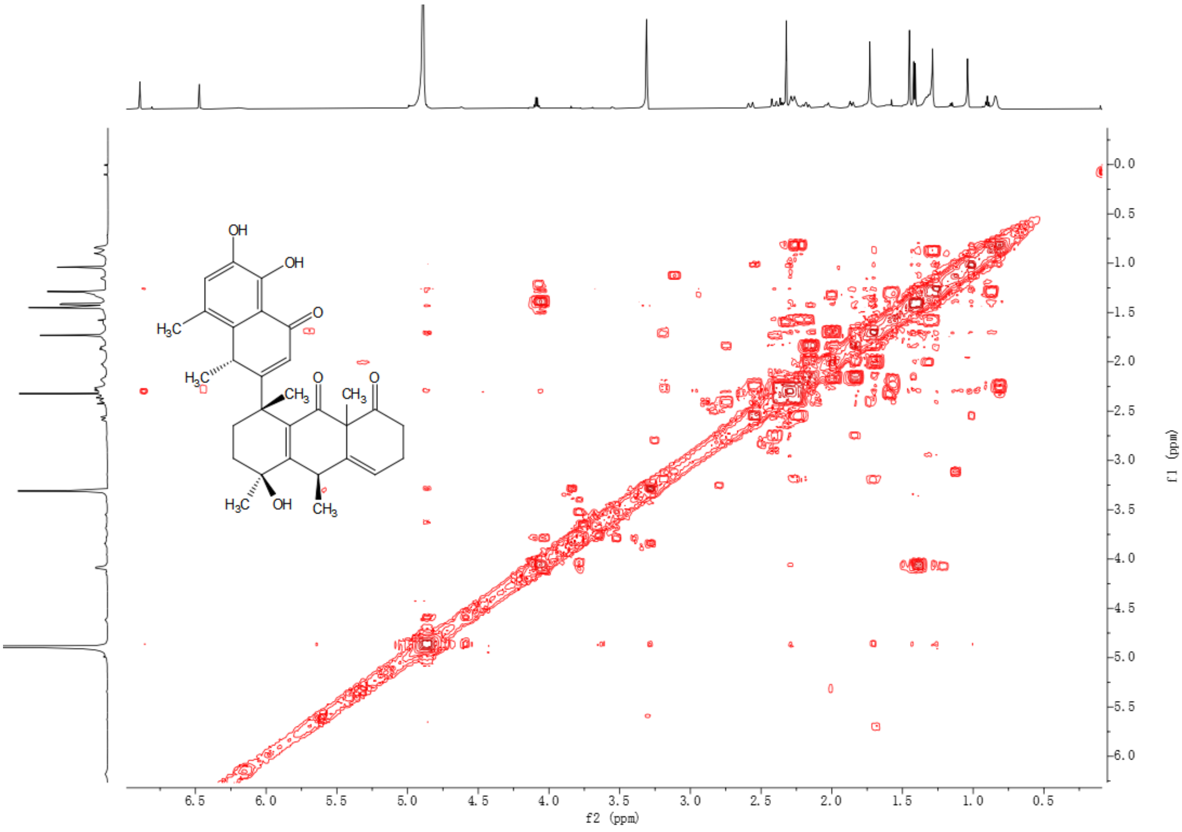

Figure S20: ^{1}H-^{1}H COSY spectrum of compound **3** in CD_3OD

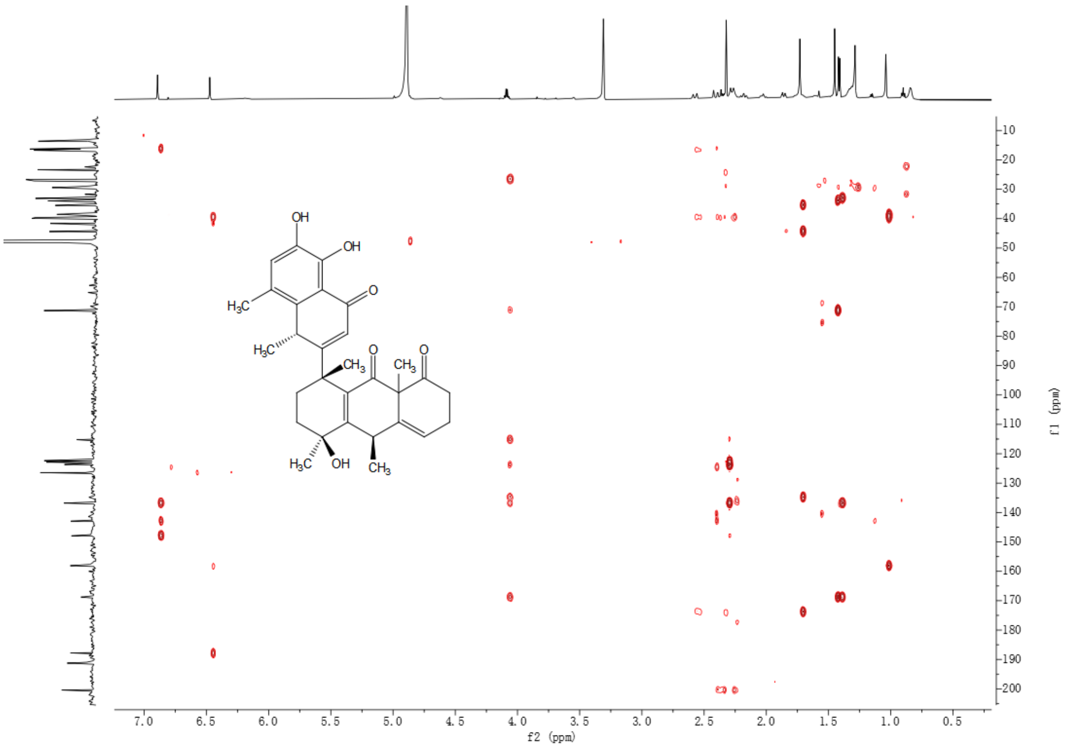

Figure S21: HMBC spectrum of compound **3** in CD_3OD

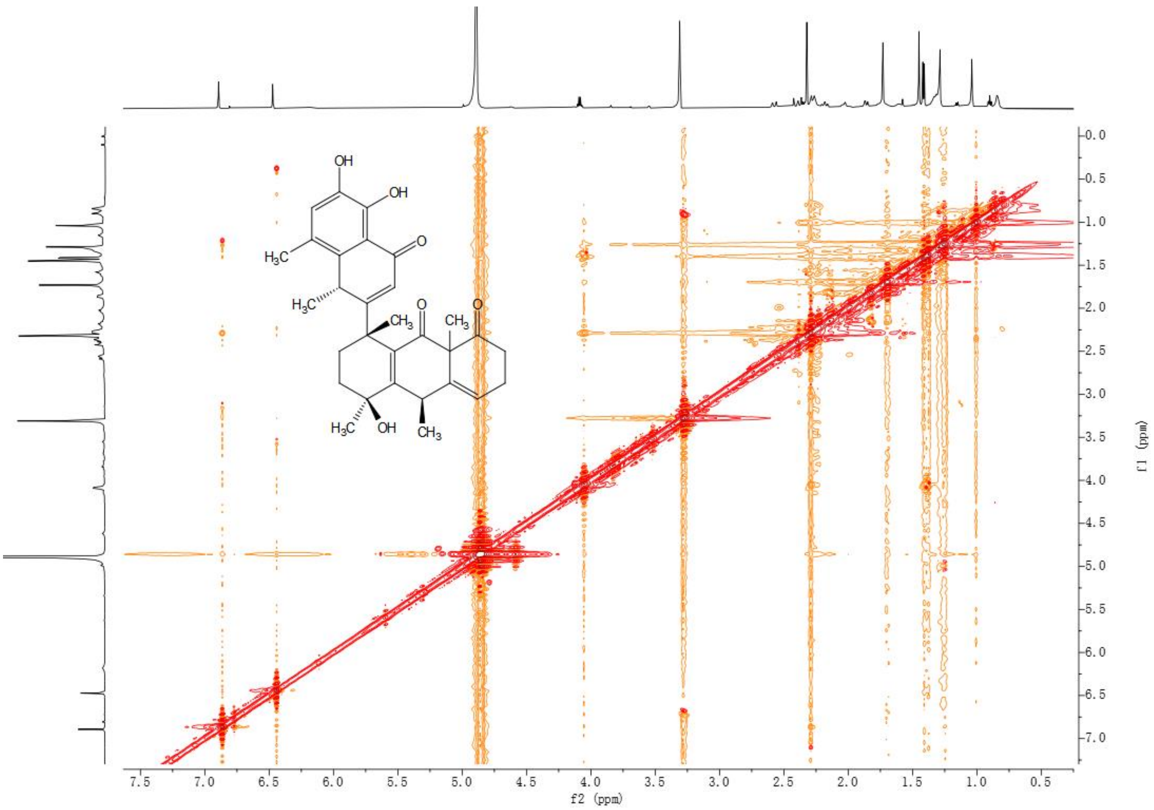

Figure S22: ROESY spectrum of compound **3** in CD_3OD

Chemical structure, and NMR date of Compound 4

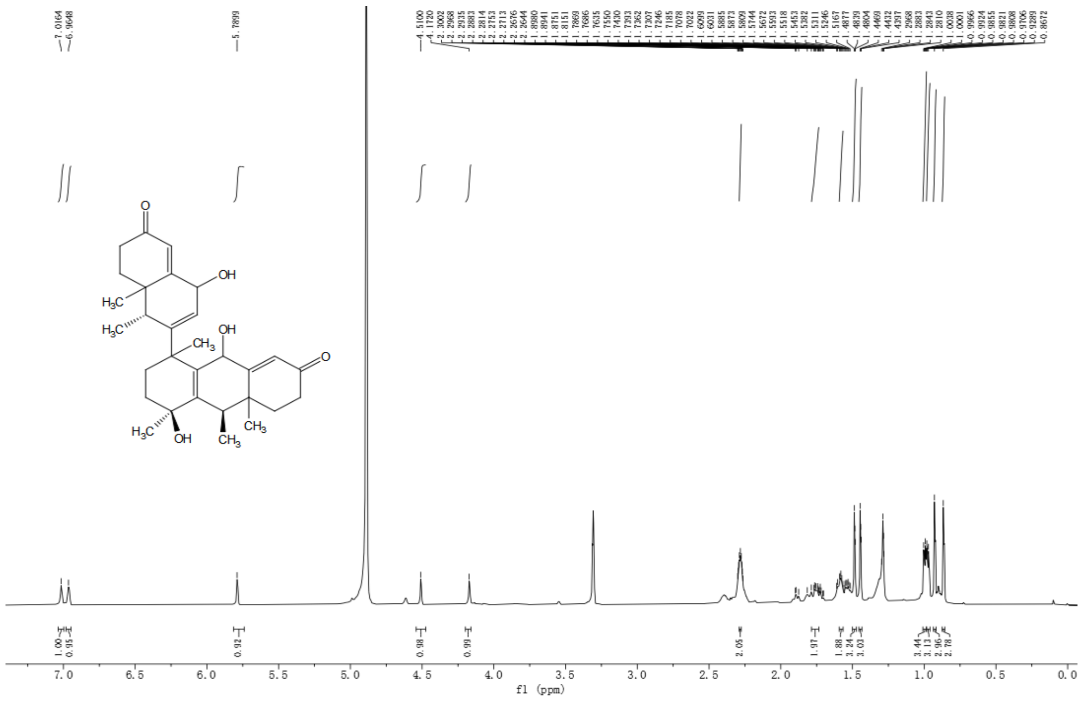

Figure S23: ^{1}H NMR spectrum of compound **4** in CD_3OD

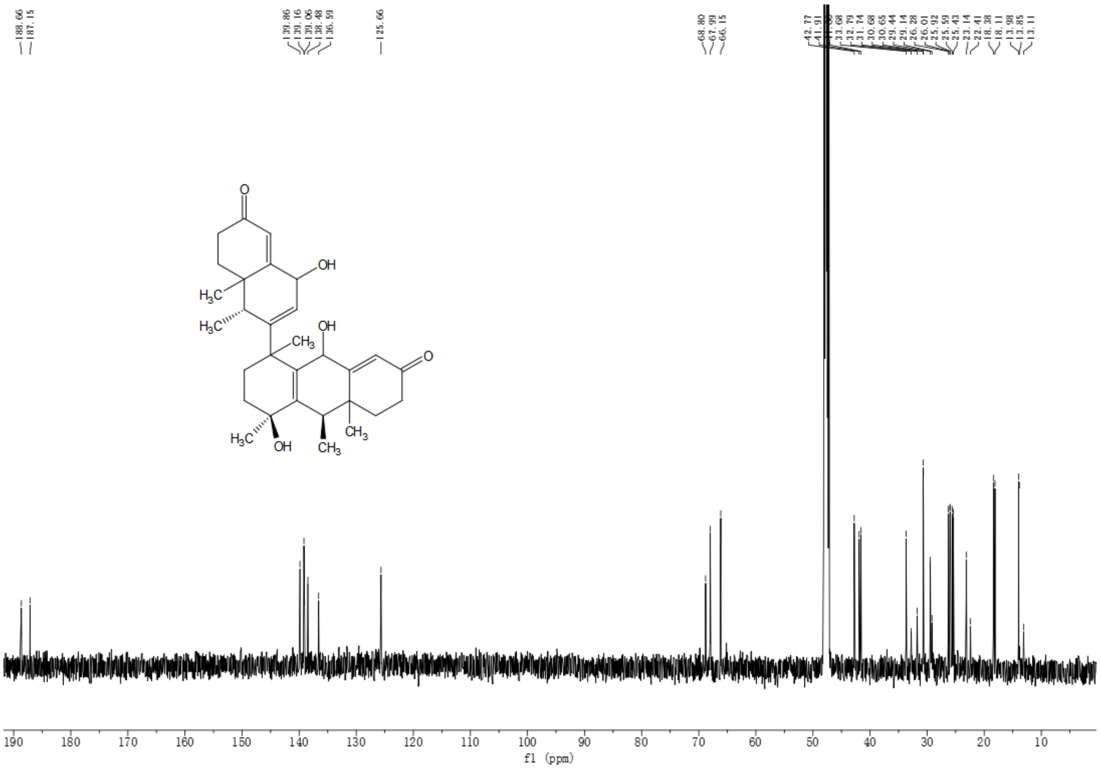

Figure S24: ^{13}C NMR spectrum of compound **4** in CD_3OD

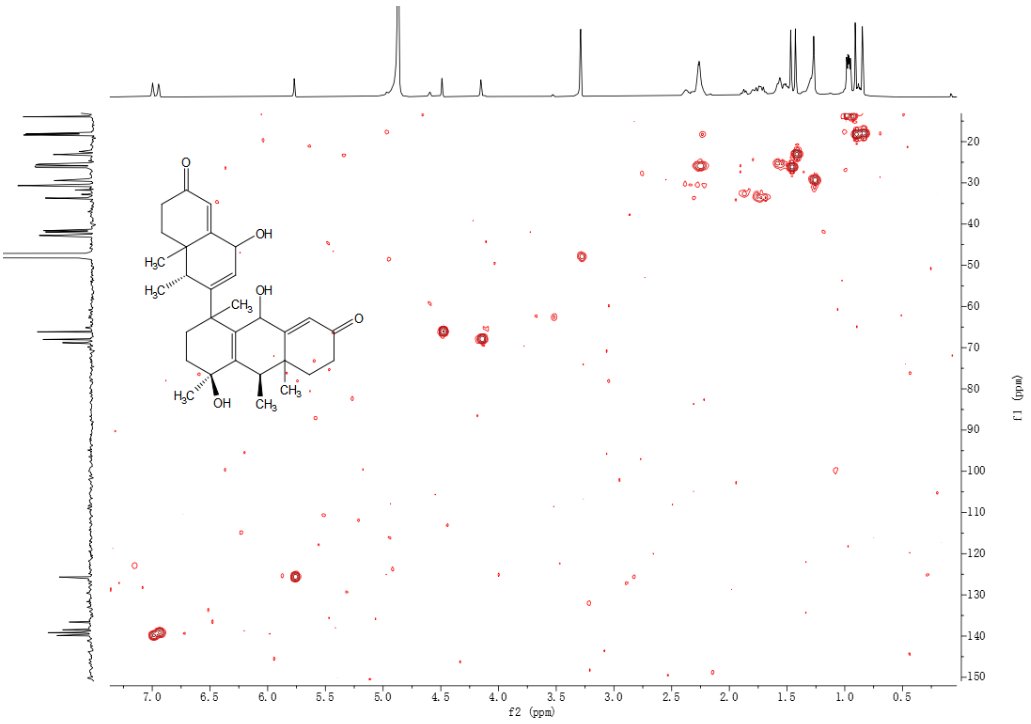

Figure S25: HMQC spectrum of compound **4** in CD_3OD

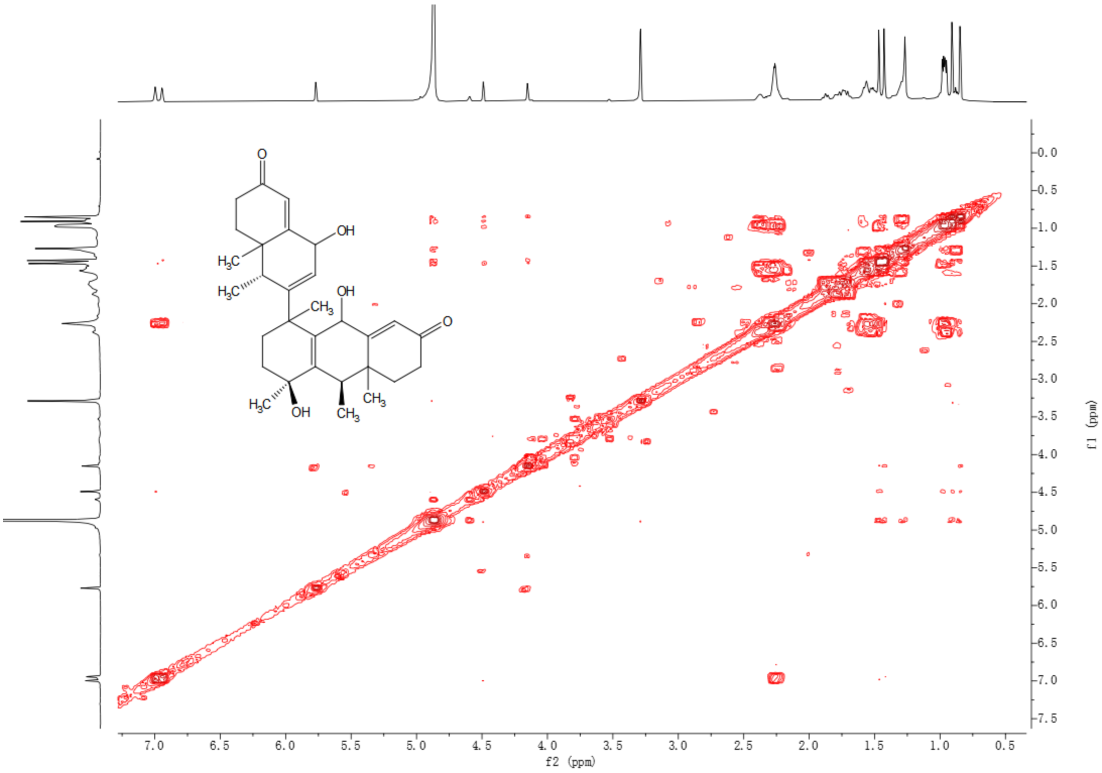

Figure S26: ^1H-^1H COSY spectrum of compound **4** in CD_3OD

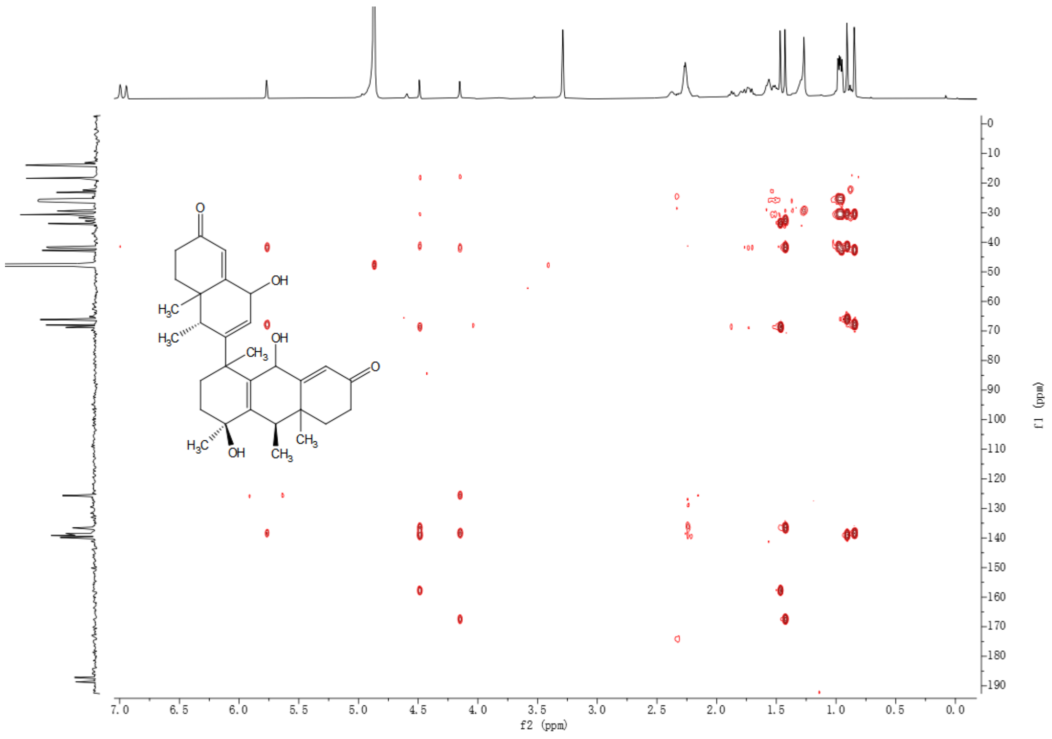

Figure S27: HMBC spectrum of compound **4** in CD_3OD

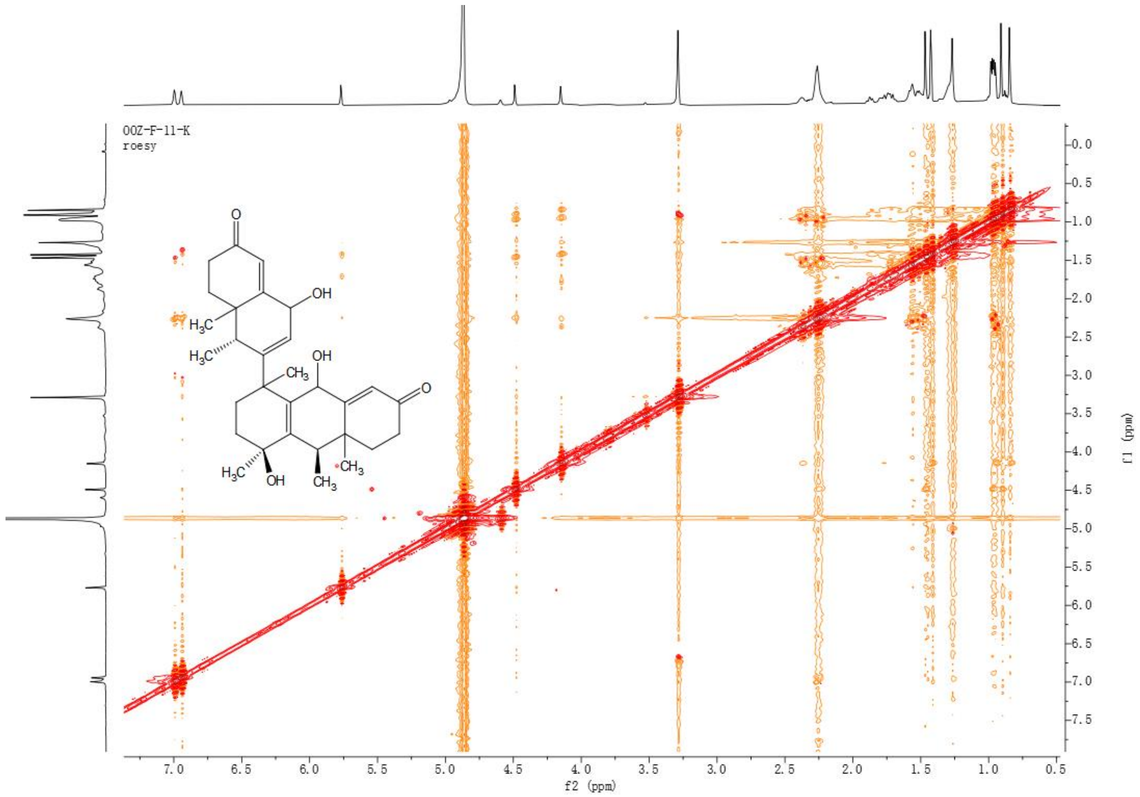

Figure S28: ROESY spectrum of compound **4** in CD_3OD

HPLC analysis of compounds **1-4**

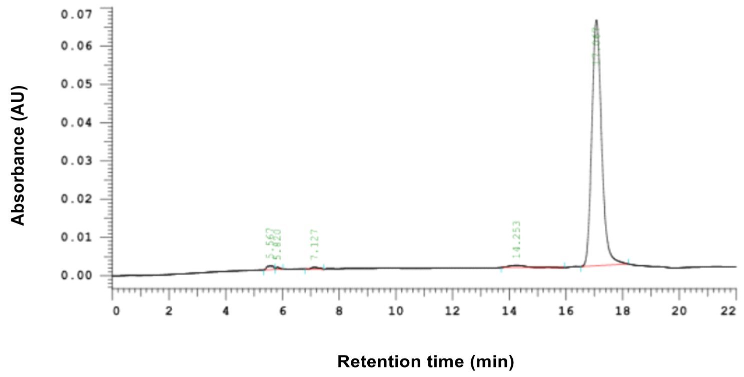

No.	RT	Peak area	Concentrations	BC
1	5. 567	7744	0. 969	BV
2	5. 820	2253	0. 282	VB
3	7. 127	5048	0. 632	BB
4	14. 253	12169	1. 522	BB
5	17. 067	772238	96. 596	BB
		799452	100. 000	

Figure S29: HPLC analysis of compound **1**

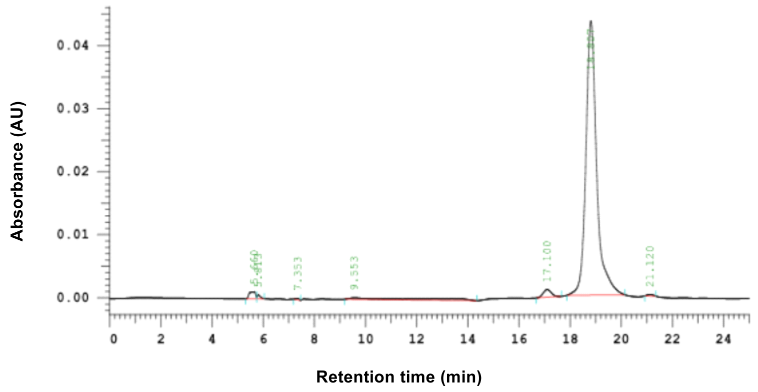

No.	RT	Peak area	Concentrations	BC
1	5. 660	9182	1. 335	BV
2	5. 813	2588	0. 376	VB
3	7. 353	1032	0. 150	BB
4	9. 553	24925	3. 625	BB
5	17. 100	13400	1. 949	BB
6	18. 807	634685	92. 295	BB
7	21. 120	1853	0. 269	BB
		687665	100. 000	

Figure S30: HPLC analysis of compound **2**

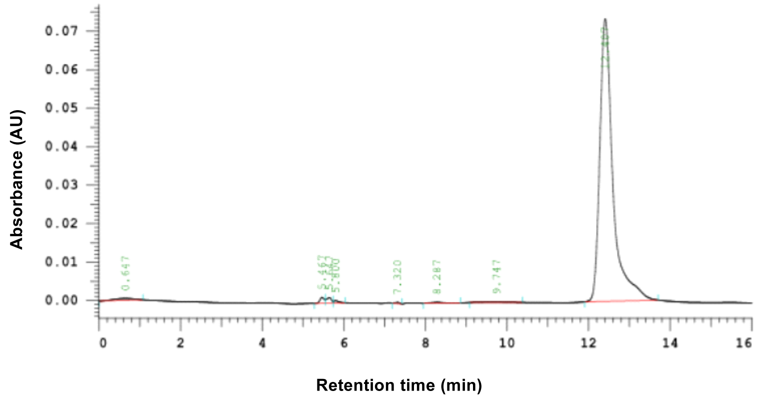

No.	RT	Peak area	Concentrations	BC
1	0. 647	8349	0. 985	BB
2	5. 467	5289	0. 624	BV
3	5. 647	5976	0. 705	VV
4	5. 800	2601	0. 307	VB
5	7. 320	1018	0. 120	BB
6	8. 287	1759	0. 208	BB
7	9. 747	3243	0. 383	BB
8	12. 407	819275	96. 668	BB
		847510	100. 000	

Figure S31: HPLC analysis of compound **3**

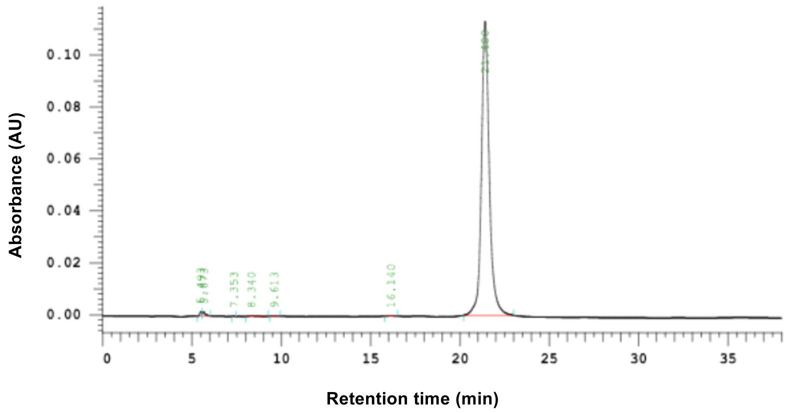

No.	RT	Peak area	Concentrations	BC
1	5. 493	6090	0. 349	BV
2	5. 673	8747	0. 502	VB
3	7. 353	1227	0. 070	BB
4	8. 340	2128	0. 122	BB
5	9. 613	846	0. 049	BB
6	16. 140	1422	0. 082	BB
7	21. 400	1723197	98. 827	BB
		1743657	100. 000	

Figure S32: HPLC analysis of compound **4**

HRESIMS results of compounds 1 to 4

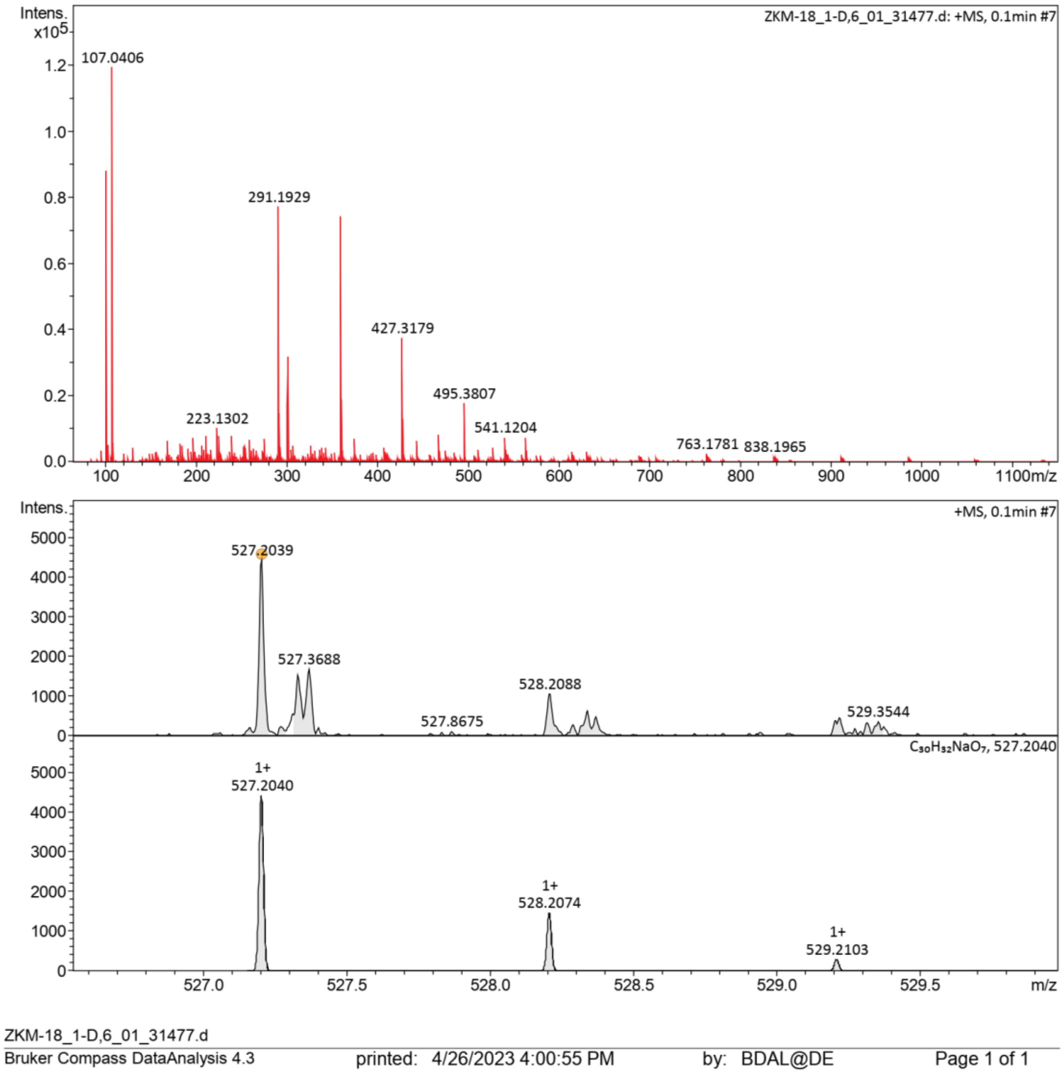

Figure S33: HRESIMS of compound **1**

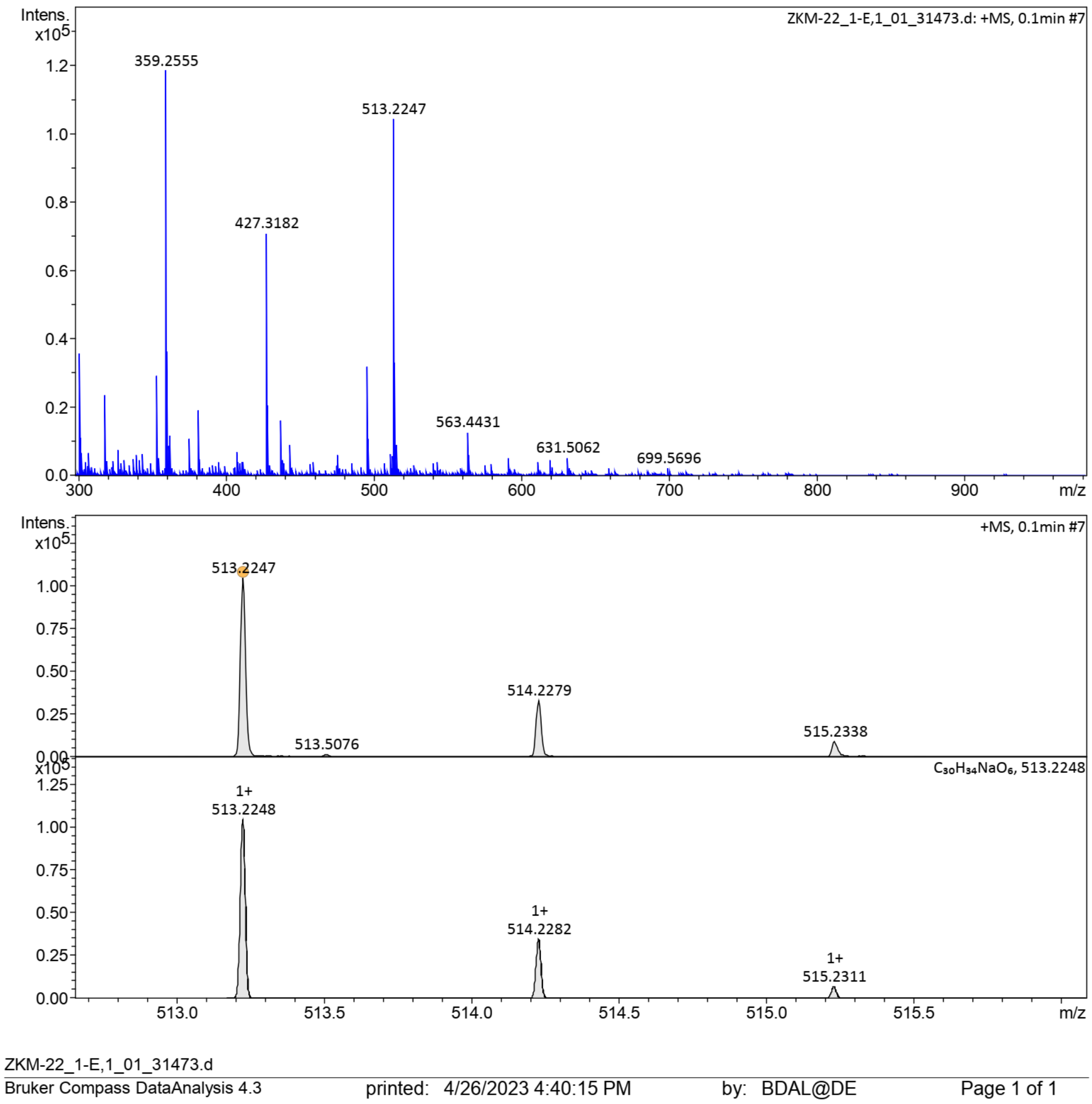

Figure S34: HRESIMS of compound **2**

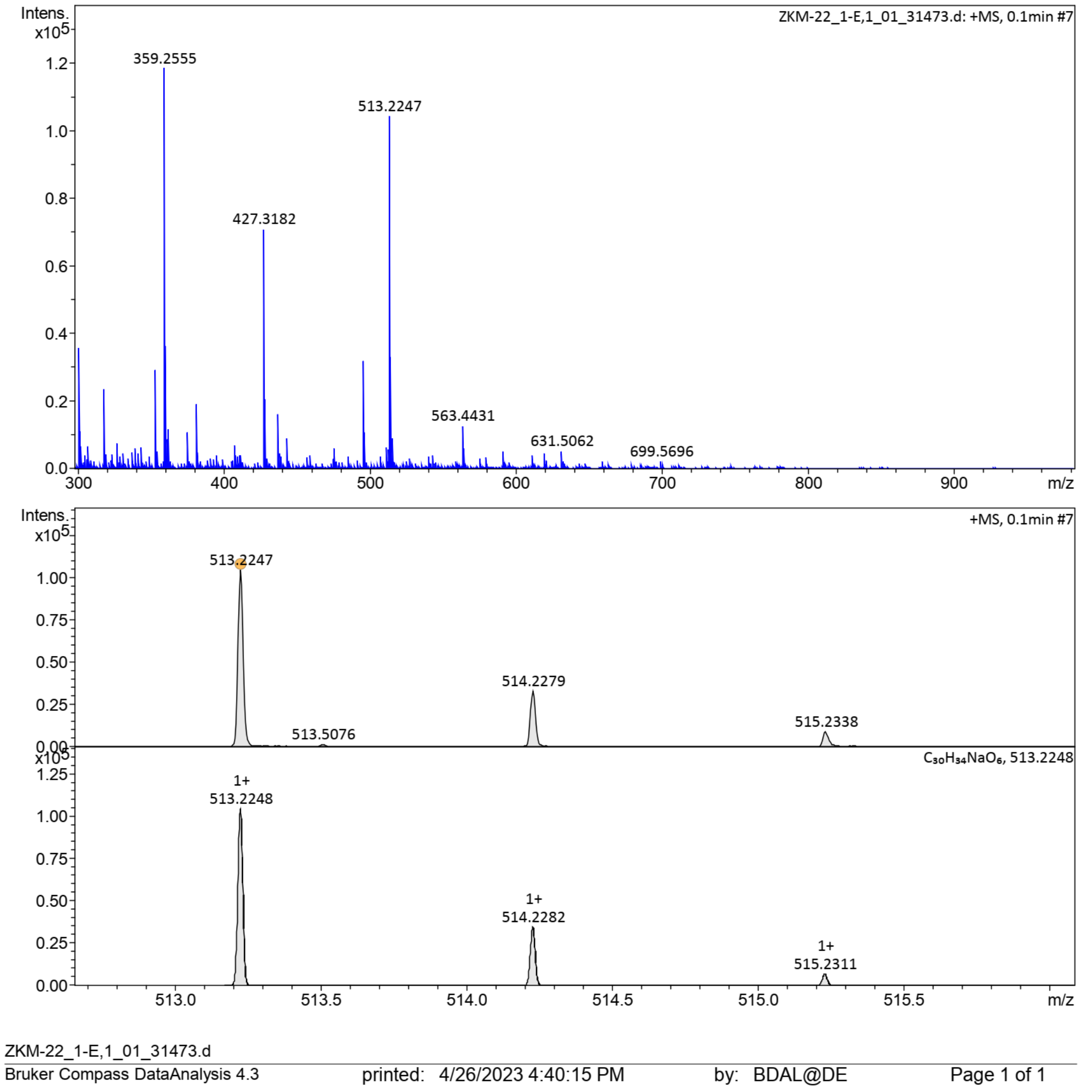

Figure S35: HRESIMS of compound **3**

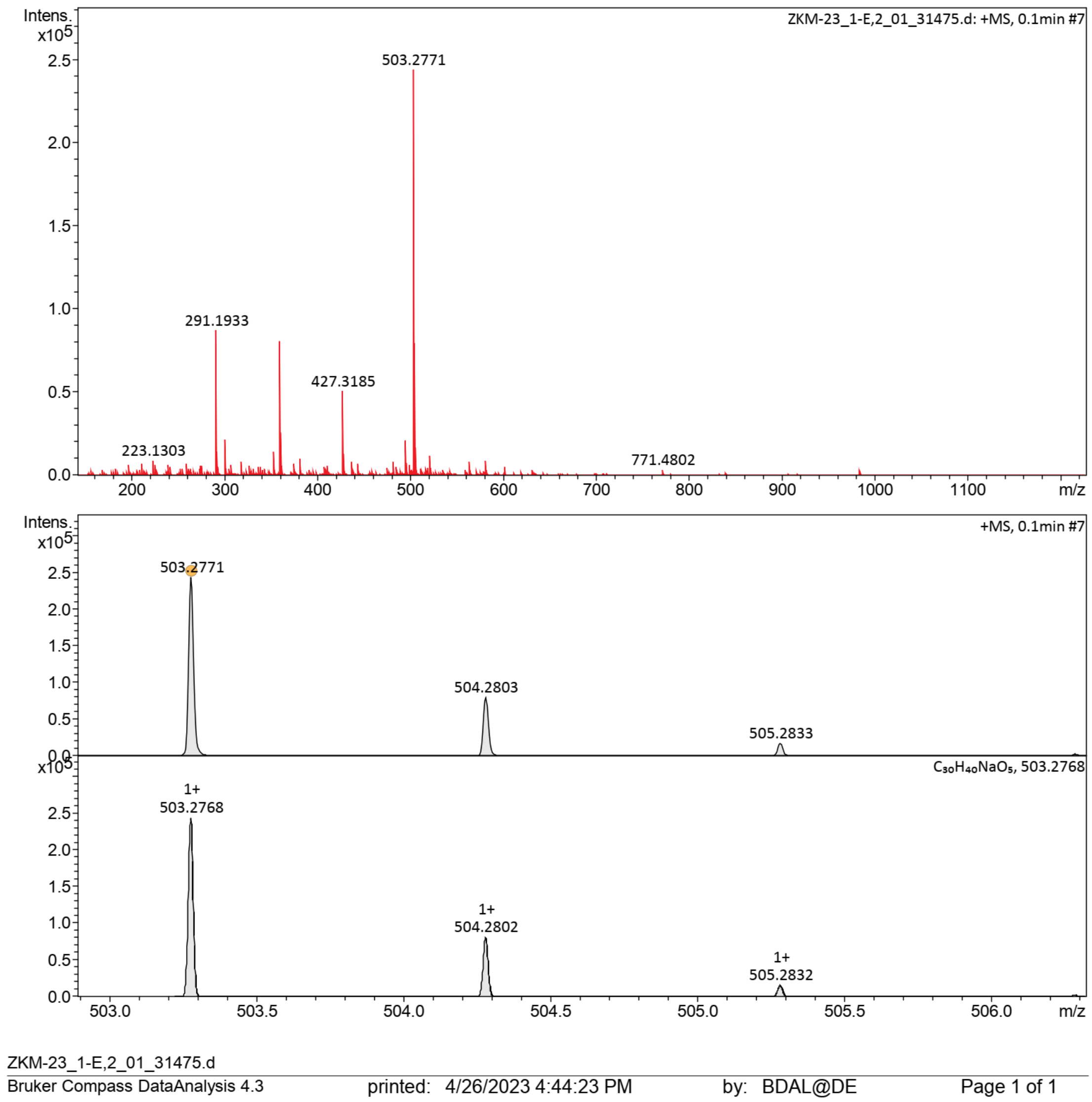

Figure S36: HRESIMS of compound **4**
